# The Antioxidant Gallic Acid Inhibits Aflatoxin Formation in *Aspergillus flavus* by Modulating Transcription Factors FarB and CreA

**DOI:** 10.3390/toxins10070270

**Published:** 2018-07-03

**Authors:** Xixi Zhao, Qing-Qing Zhi, Jie-Ying Li, Nancy P. Keller, Zhu-Mei He

**Affiliations:** 1 The Guangdong Province Key Laboratory for Aquatic Economic Animals, School of Life Science, Sun Yat-sen University, Guangzhou 510275, China; zhaoxifdu@163.com (X.Z.); zhiqq3@mail.sysu.edu.cn (Q.-Q.Z.); lijieyingjy@126.com (J.-Y.L.); 2 Department of Medical Microbiology and Immunology, University of Wisconsin-Madison, Madison, WI 53706, USA

**Keywords:** *Aspergillus flavus*, antioxidant gallic acid, aflatoxin, *farB*, *creA*

## Abstract

Aflatoxin biosynthesis is correlated with oxidative stress and is proposed to function as a secondary defense mechanism to redundant intracellular reactive oxygen species (ROS). We find that the antioxidant gallic acid inhibits aflatoxin formation and growth in *Aspergillus flavus* in a dose-dependent manner. Global expression analysis (RNA-Seq) of gallic acid-treated *A. flavus* showed that 0.8% (*w*/*v*) gallic acid revealed two possible routes of aflatoxin inhibition. Gallic acid significantly inhibited the expression of *farB*, encoding a transcription factor that participates in peroxisomal fatty acid β-oxidation, a fundamental contributor to aflatoxin production. Secondly, the carbon repression regulator encoding gene, *creA*, was significantly down regulated by gallic acid treatment. CreA is necessary for aflatoxin synthesis, and aflatoxin biosynthesis genes were significantly downregulated in ∆*creA* mutants. In addition, the results of antioxidant enzyme activities and the lipid oxidation levels coupled with RNA-Seq data of antioxidant genes indicated that gallic acid may reduce oxidative stress through the glutathione- and thioredoxin-dependent systems in *A. flavus*.

## 1. Introduction

*Aspergillus flavus* is not only a saprotrophic and plant pathogenic fungus but also an opportunistic human and animal pathogen [1]. *A. flavus* is notorious for production of aflatoxins, which were discovered as the main cause of Turkey-X disease in the 1960s [2]. Since then, substantial efforts have been directed toward understanding the complex mechanisms and the regulation network of aflatoxin biosynthesis.

Primary metabolism has a close link with secondary metabolite synthesis in fungi and, in the case of aflatoxin, several studies have shown that β-oxidation contributes to high levels of aflatoxin. Fatty acid β-oxidation in both the peroxisome and mitochondria has a fundamental contribution to toxin production, such as the polyketides, aflatoxin, and sterigmatocystin [3]. Reverberi et al. treated *A. flavus* with bezafibrate, an inducer of peroxisomal β-oxidation in mammals, and stimulated aflatoxin production. They also introduced a P33 gene into *A. flavus* to induce peroxisome proliferation, which leads to an upregulation of lipid metabolism and higher content of intracellular reactive oxygen species (ROS), with increasing aflatoxin formation [4]. The metabolomic studies also demonstrated that the lack of aflatoxin formation in a known aflatoxin inhibitory medium (peptone) was accompanied with suppressed fatty acid synthesis and reduced tricarboxylic acid (TCA) cycle intermediates, and increased pentose phosphate pathway products [5]. The accumulating NADPH pool, derived primarily from the pentose phosphate pathway, was suggested to suppress aflatoxin synthesis by directing the acyl-CoA into lipid synthesis, rather than polyketide biosynthesis [6,7]. 

In addition to a requirement for adequate acyl-CoA pools to synthesize aflatoxin, several groups have proposed that oxidative stress is a prerequisite for aflatoxin biosynthesis in *Aspergillus parasiticus* [8,9,10]. This observation is tied in with the hypothesis that aflatoxin is a secondary defense system protecting the fungus from excess ROS [11]. Supporting these proposals are studies showing that compounds which altered oxidative stress in *A. flavus* or *A. parasiticus* also affected aflatoxin production. For example, the pro-oxidants cumene, hydroperoxide, and hydrogen peroxide promoted aflatoxin biosynthesis in *A. flavus* [4], and epoxides stimulated aflatoxin formation by increasing the lipid peroxidation in *A. flavus* and *A. parasiticus* [12]. Conversely, antioxidants, such as ethylene, alleviated the oxidative stress and changed the glutathione redox state in *A. flavus*, which resulted in inhibiting aflatoxin biosynthesis [13]. *Lentinula edodes* β-glucan significantly reduced aflatoxin formation in *A. parasiticus* by stimulating antioxidant enzyme activity, and the activation of antioxidant response-related transcription factors, which correlated with a delay of aflatoxin genes expression [14]. Piperine inhibited aflatoxin production in *A. flavus* concurrently with positive regulation of genes belonging to superoxide dismutase and catalase families, as well as genes encoding the basic leucine zipper (bZIP) transcription factors AtfA, AtfB, and Ap-1 [15]. These bZIP transcription factors and MsnA are thought to participate in a regulatory network that mediates both the oxidative stress and aflatoxin pathways in *A. parasiticus* [11].

Gallic acid (GA), a constituent in the pellicle of Tulare walnut, has shown potent inhibitory activity toward aflatoxin biosynthesis [16], but the mechanism of GA inhibition of aflatoxin formation has not been studied. We present here our finding that GA significantly inhibited the expression of the *farB* gene, which controls the activity of peroxisomal fatty acid β-oxidation [17], and of the carbon repression regulator encoding gene, *creA*, which has recently been found involved in aflatoxin synthesis [18]. Simultaneously, the expression of almost all assigned genes in the aflatoxin biosynthesis cluster was significantly downregulated by 0.8% (*w*/*v*) GA treatment. Our work provides insights into cellular mechanisms underlying oxidative stress responses contributing to aflatoxin biosynthesis in *A. flavus*.

## 2. Results

### 2.1. Effect of GA on Aflatoxin Biosynthesis and Growth in A. flavus

**Figure 1:**
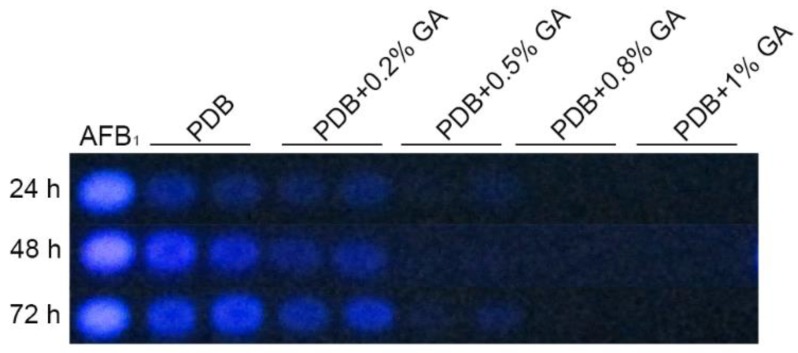
Gallic acid (GA) inhibits aflatoxin biosynthesis in *Aspergillus flavus* in a dose-dependent manner.

**Figure 2:**
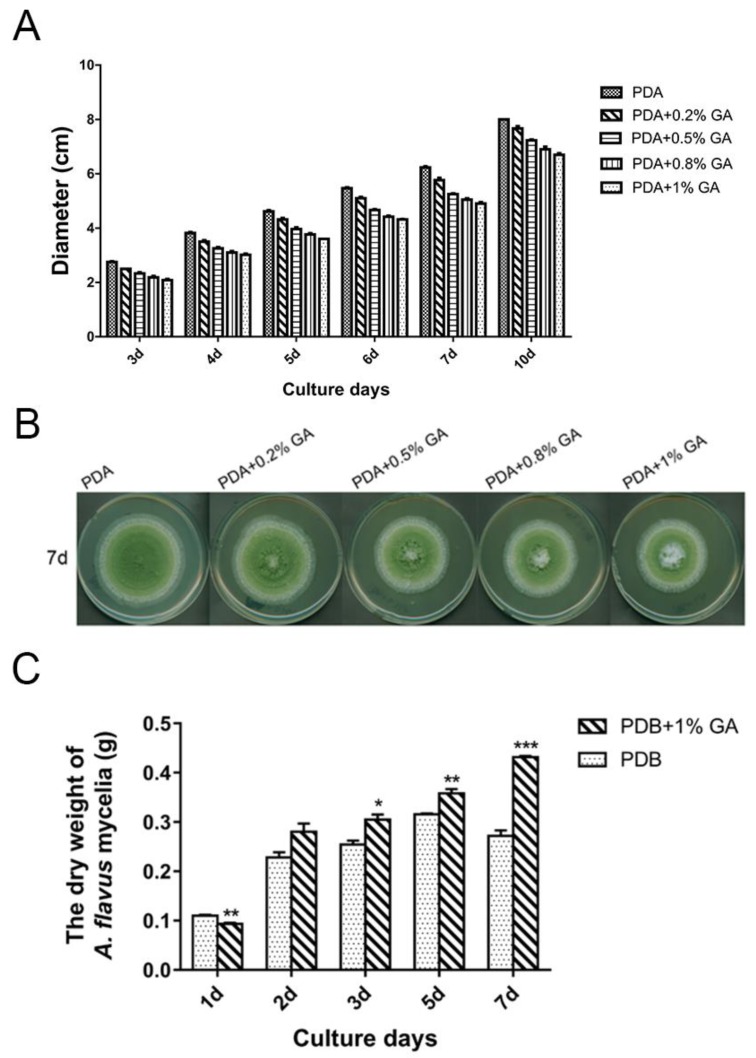
GA affects *A. flavus* growth. (**A**) 10^3^ spores were inoculated on PDA medium with different concentrations (*w*/*v*) of GA and cultured at 30 °C, the diameters were measured on different culture days. (**B**) The photo of the samples on the 7th day cultivation. (**C**) 10^7^ spores were inoculated into 30 mL PDB with or without 1% (*w*/*v*) GA and cultured at 200 rpm, 30 °C for different days, then the mycelia were collected and measured. Each treatment has three replicates, *: *p* < 0.05, **: *p* < 0.01, ***: *p* < 0.001.

Treatment of *A. flavus* NRRL3357 spores with different concentrations (0, 0.2%, 0.5%, 0.8%, and 1%, *w*/*v*: Weight/volume) of GA showed that GA inhibited aflatoxin B_1_ synthesis in a dose-dependent manner (Figure 1). Aflatoxin B_1_ production was significantly inhibited when the GA concentration was 0.5% (*w*/*v*), and was totally inhibited with greater than 0.8% (*w*/*v*) GA in the medium. Aflatoxin reduction was positively correlated with a decrease in *A. flavus* colony diameter (Figure 2A,B), with the maximum inhibition rate of 24% in the 1% (*w*/*v*) treated samples at the 3rd-day cultivation (Table S1). However, when *A. flavus* was inoculated into liquid PDB medium, mycelial mass increased in the 1% (*w*/*v*) GA treated samples compared with the untreated samples (Figure 2C). 

A sample of 10^7^ spores was inoculated into 30 mL PDB with different concentration (0, 0.2%, 0.5%, 0.8%, 1%, *w*/*v*) of GA, cultured at 30 °C, 200 rpm. Next, 200 µL culture medium were taken out at 24 h, 48 h, and 72 h, respectively, for TLC analysis, AFB_1_: Aflatoxin B_1_ standard from Sigma. These were 0.2% GA: 0.2% (*w*/*v*) GA, 0.5% GA: 0.5% (*w*/*v*) GA, 0.8% GA: 0.8% (*w*/*v*) GA, 1% GA: 1% (*w*/*v*) GA. *w*/*v*: Weight/volume, such as 0.3 g GA add into 30 mL PDB medium will make a PDB+1% (*w*/*v*) GA.

### 2.2. Transcriptomic Profiles of A. flavus Responding to GA

**Figure 3:**
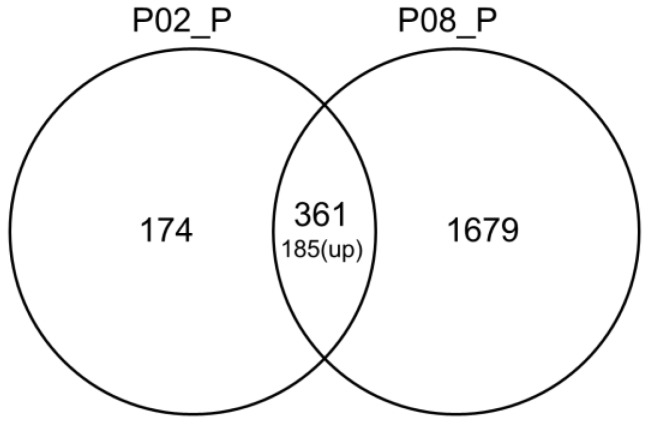
Venn diagram of the significantly differentially expressed genes. In total, 361 genes were shared in these two sets, of which 185 were upregulated. P02_P: Significantly differentially expressed genes between 0.2% (*w*/*v*) gallic acid-treated and untreated *A. flavus* samples. P08_P: Significantly differentially expressed genes between 0.8% (*w*/*v*) gallic acid-treated and untreated *A. flavus* samples.

In order to gain insight into the mechanism by which GA inhibits aflatoxin synthesis, RNA was extracted from 24 h cultures treated with either 0.2% (*w*/*v*) GA (partly inhibits aflatoxin B_1_ formation) or 0.8% (*w*/*v*) GA (totally inhibits aflatoxin B_1_ formation) for RNA-Seq analysis. Table S2 summarizes the RNA-Seq data. The FPKM [19] value had been applied to quantify the level of each genes’ expression, and the count number of each gene from the three sequenced samples were analyzed by DEseq software [20] for genes’ different expression analysis. The genes with |log2Ratio| ≥ 1 and *q* < 0.05 were considered as significantly differentially expressed and listed in Table S3. In total, 535 genes were significantly differentially transcribed between 0.2% (*w*/*v*) GA treated and untreated *A. flavus* samples, and 2040 significantly differentially expressed genes were found in 0.8% (*w*/*v*) GA treated compared with untreated *A. flavus* samples (Table 1). In addition, 361 shared genes were found in these two sets (Figure 3, Table S3).

### 2.3. Effect of GA on the Expression of Genes in A. flavus Secondary Metabolism Clusters 

**Figure 4:**
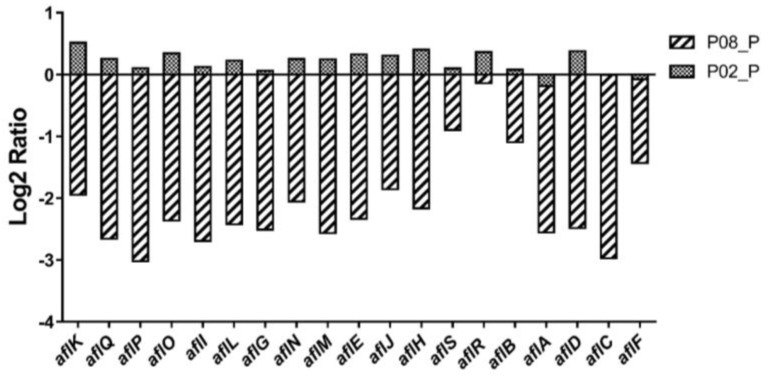
Effect of GA on the expression of the aflatoxin assigned genes in *A. flavus*. P: Untreated *A. flavus* samples, P02: 0.2% (*w*/*v*) GA treated *A. flavus* samples, P08: 0.8% (*w*/*v*) GA treated *A. flavus* samples. P02_P Ratio: The mean value of the count number of each gene in P02/in P. P08_P Ratio: The mean value of the count number of each gene in P08/in P.

There are 56 predicted secondary metabolism gene clusters in *A. flavus* [21,22], most of which contain a gene encoding for a ‘backbone’ enzyme (e.g., PKS, NRPS, DMATs, terpene, etc) essential for their respective secondary metabolite biosynthesis [22]. The effect of GA on the *A. flavus* 56 secondary metabolism clusters activity was analyzed based on the ‘backbone’ genes expression in our RNA-Seq data, and the data showed that there were 16 secondary metabolism clusters significantly affected by GA treatment (Table S4). The cluster #54, responsible for the aflatoxin biosynthesis, was one of these 16 clusters, and almost all the assigned genes (except the regulatory genes *aflR* and *aflS* [23]) expression were significantly downregulated in the 0.8% (*w*/*v*) GA treated samples compared with the untreated samples (Figure 4, Table S3). These data are consistent with the 0.8% (*w*/*v*) GA totally inhibiting aflatoxin formation in *A. flavus* (Figure 1).

### 2.4. Expression Changes of the Developmental Regulatory Genes and the Primary Metabolic Genes in A. flavus in Response to GA

Because the genes encoding aflatoxin transcription factors *aflR* and *aflS* were not significantly affected by GA (Table S3), we next examined the RNA-seq for other clues as to why aflatoxin biosynthesis and gene expression were down regulated. We first looked at transcriptional regulators of aflatoxin. The Velvet Complex heterotrimer is a critical transcriptional complex required for secondary metabolism and fungal development in *Aspergillus* spp. [24], including *A. flavus* [25]. However, in our data, the expression of all three members, *veA, velB*, and *laeA* were not significantly different in the GA-treated *A. flavus* samples compared with the untreated *A. flavus* samples. We also observed that *nsdC* encoding a C_2_H_2_ zinc finger-type DNA-binding protein, which is required for normal sclerotia formation and aflatoxin biosynthesis in *A. flavus* [26], was upregulated (Table 2) and thus not a likely cause of loss of aflatoxin production by GA.

Carbon utilization pathways have also been associated with aflatoxin biosynthesis. In our RNA-Seq data, most of the putative genes responsible for the pentose phosphate pathway were upregulated by GA treatment, such as AFLA_086620, AFLA_080390, and AFLA_036840 (Table 2) encoding proteins homologous to Zwf1, Sol3, and Gnd1 in *Candida albicans*, respectively [27]. Pentose phosphate pathway activity has been linked to decreased aflatoxin synthesis in a previous study [5] and thus supports a possible role of this pathway in gallic acid repression of aflatoxin production. 

Fatty acids and β-oxidation have been largely shown to promote aflatoxin biosynthesis [3,28]. The most striking finding from the RNA-seq data was the large number of peroxisome and fatty acid β-oxidation-related genes (Table S5) which were significantly and primarily down regulated by GA treatment based on the GO analysis (Table S6). These genes included the peroxisomal β-oxidation multifunctional enzyme encoding gene, *foxA* [29], and acetyl-carnitine transferase encoding gene, *acuJ* [30]. The fatty acid metabolism regulatory transcription factor gene, *farB* (AFLA_012010), was also downregulated. FarB has been reported as positively affecting aflatoxin biosynthesis in *A. flavus* via regulating the expression of the peroxisomal fatty acid β-oxidation-related genes *pexK*, *foxA*, and *acuJ* [17].

**Figure 5:**
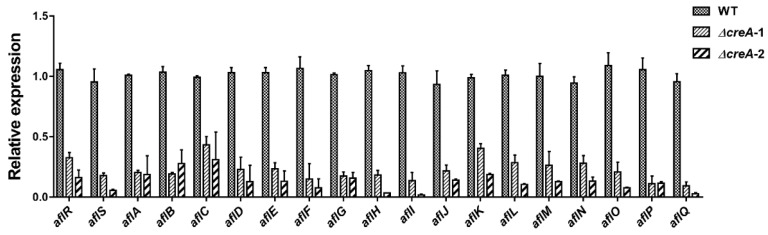
Expression ratios of the aflatoxin biosynthesis-assigned genes in the ∆*creA* mutants compared to wild type WT(TJW149.27). A total of 10^7^ spores of WT, ∆*creA*-1, and ∆*creA*-2 were inoculated into 30 mL PDB medium, and cultured at 200 rpm, 30 °C for 3 days, the mycelia was collected for RNA extraction, then the RNA was reverse transcript into cDNA for qRT-PCR analysis. The primers for the qRT-PCR can be found in Table S7. The expression of each gene in the WT was normalized as 1.0, each sample has three replicates, and the error bars indicate the standard error of the mean.

Finally, we noted that the carbon catabolite repression gene, *creA* [31], decreased with the increasing concentration of GA and was significantly downregulated in the 0.8% (*w*/*v*) GA-treated samples compared with the untreated samples (Table 2). CreA regulates gene expression, either directly by binding to the consensus binding sites (5′-SYGGRG-3′) in the promoter of the target genes [32], or through transcriptional cascades. This consensus binding site is found in 14 out of the 19 aflatoxin gene promoter regions (Table 3), and because expression of these genes (except *aflR* and *aflS*) was significantly downregulated by GA treatment, it appeared likely that GA regulation of *creA* was also contributing to the reduction of aflatoxin synthesis. Indeed we found that all 19 assigned aflatoxin genes were significantly downregulated in two independent ∆*creA* mutants compared with wild type (WT) (Figure 5). These results were consistent with the loss of aflatoxin synthesis in the deletion of *creA* in another strain of *A. flavus*, CA14 [18]. 

### 2.5. Effect of GA on Oxidative Stress Genes and the Antioxidant Enzymatic Activities

In *A. flavus*, there are several oxidative stress-related transcription factors that mediate oxidative stress and aflatoxin formation, and among these AtfA, AtfB, Ap-1, and MsnA have been characterized [33,34,35,36]. The expression of the genes encoding these transcription factors did not show significant difference in the GA-treated samples compared with the untreated samples. However, we found some of the enzymatic genes regulated by these transcription factors were differentially regulated, including the genes encoding the antioxidant enzymes catalase (AFLA_056170, AFLA_122110, and AFLA_090690) and superoxide dismutase (SOD, AFLA_099000, and AFLA_033420), which were slightly upregulated in the 0.8% (*w*/*v*) GA-treated samples compared with the untreated samples. Additionally, genes encoding the glutathione S-transferase and the thioredoxin system proteins presented significantly higher expression levels in the 0.8% (*w*/*v*) GA-treated samples compared with the untreated samples (Table 4). Oxylipin synthesis is associated with oxidative processes and aflatoxin biosynthesis and, as shown in Table 4, the *ppoC* gene encoding for oxylipin biosynthesis [37] and the GPCRs genes *gprC*, *gprK*, and *gprM*—implicated in oxylipin perception [38]—were significantly inhibited by at least one GA treatment.

**Figure 6:**
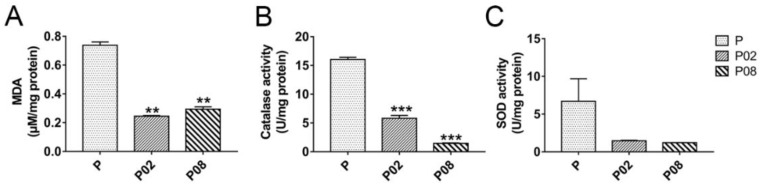
Effect of GA on lipid oxidant (**A**) and antioxidant enzymes activities (**B**,**C**) in *A. flavus*. P: Untreated *A. flavus* samples, P02: 0.2% (*w*/*v*) GA-treated *A. flavus* samples, P08: 0.8% (*w*/*v*) GA-treated *A. flavus* samples. **: *p* < 0.01, ***: *p* < 0.001. MDA = Malondialdehyde, SOD = super oxide dismutase.

To gain more information on this possible linkage of GA with oxidative processes, the activities of catalase and SOD and the level of Malondialdehyde (MDA, as a marker of lipid oxidation) were measured in the 0.2% (*w*/*v*) and 0.8% (*w*/*v*) GA-treated and untreated *A. flavus* samples with 24 h cultivation. Results, shown in Figure 6, demonstrated that the MDA content was decreased by GA treatment (Figure 6A), and the activities of catalase and SOD were also inhibited in the GA treated samples compared with the untreated samples (Figure 6B,C), despite a slight increase in gene expression. These results and the expression of the antioxidant genes indicate that GA may balance oxidative stress homeostasis in *A. flavus* via activating the glutathione- and thioredoxin-dependent antioxidant system, rather than through catalase and SOD activity.

## 3. Discussion

Upon finding that GA was effective in suppressing aflatoxin synthesis, Mahoney and Molyneux [16] suggested the mechanism might be through GA control of the fatty acid synthases (*aflA*, *aflB*) or polyketide synthase (*aflC*) required for generation of norsolorinic acid, the first visible and stable intermediate in the aflatoxin synthesis pathway [23], which accumulates in peroxisome [3]. In this study, we not only observe down-regulation of these genes, but also identify the cellular pathways which may be affecting regulation of these genes and aflatoxin synthesis in general. We demonstrated that GA inhibited aflatoxin biosynthesis in *A. flavus* in a dose-dependent manner, where aflatoxin formation was partly inhibited by 0.2% (*w*/*v*) GA treatment and totally inhibited by more than 0.8% (*w*/*v*) GA treatment. To find out the genes mediating GA and aflatoxin biosynthesis in *A. flavus*, the transcriptomic profile of the 0, 0.2%, and 0.8% (*w*/*v*) GA-treated *A. flavus* samples was obtained based on the RNA-Seq technology. Taken together, the transcriptional profiling results suggested GA could be suppressing aflatoxin synthesis through several cellular pathways that have already been implicated in aflatoxin regulation: One inhibitory (pentose phosphate pathway) and two stimulatory routes (FarB mediated β-oxidation and CreA activity). The expression changes of those genes in 0.2% (*w*/*v*) GA treatment is consistent with the partly inhibited aflatoxin formation in this treatment. Both the pentose phosphate pathway and β-oxidation pathway are active in the peroxisome, the site of the initial steps of aflatoxin biosynthesis [3]. Despite GA’s role as an antioxidant, the RNA-seq and physiological testing (Figure 6) were less clear on the role of antioxidant pathways in suppressing aflatoxin synthesis, although potential involvement of the glutathione- and thioredoxin-dependent antioxidant systems will be a future avenue to address. We also note that the antioxidant activity of the gallic acid molecule itself could be a contributing factor [39].

Aflatoxin molecular formation needs more than 23 enzymatic reactions [23] and at least 10 NADPHs [40], however, there is a competition for NADPH in acetyl-CoA incorporation into lipid synthesis, rather than into polyketides like aflatoxin [6]. In the toxin-supporting medium (Glucose-mineral salts medium, GMS), diminished NADPH generating capacity indicated that depressed NADPH/NADP ratios favor aflatoxin biosynthesis [41]. The two dehydrogenases (Zwf1 and Gnd1) in the oxidative branch of the pentose phosphate pathway, the main source of NADPH in the cell, are located in peroxisomes in *Candida albicans* [27]. The genes AFLA_086620 and AFLA_036840, encoding the homologous Zwf1 and Gnd1 proteins, respectively, were upregulated by GA treatment (Table 2), which indicated that GA treatment might regulate *A. flavus* intracellular NADPH pools and promote acyl-CoA incorporation into lipid synthesis, rather than polyketide production. 

Acetyl-CoA is produced from the oxidative decarboxylation of pyruvate and fatty acid β-oxidation [40], and fatty acid β-oxidation is a fundamental contributor to polyketide mycotoxin (aflatoxin and sterigmatocystin) formation [3]. The fatty acid transcription factor FarB—which activates peroxisomal β-oxidation genes—has been shown to positively regulate aflatoxin production in *A. flavus* [17]. In our RNA-Seq data, most of the genes involved in fatty acid β-oxidation, including *farB*, were downregulated by GA treatment (Table S5). The transcript of *farB*, *foxA* (encoding the peroxisomal β-oxidation multifunctional enzyme), and *acuJ* (encoding carnitine acetyltransferase), were significantly decreased in the 0.8% (*w*/*v*) GA-treated samples compared to the untreated samples (Table 2). Suppression of the peroxisomal β-oxidation pathway genes by GA treatment would be expected to reduce acyl-CoA pools and inhibit aflatoxin formation through lack of precursors. 

**Figure 7:**
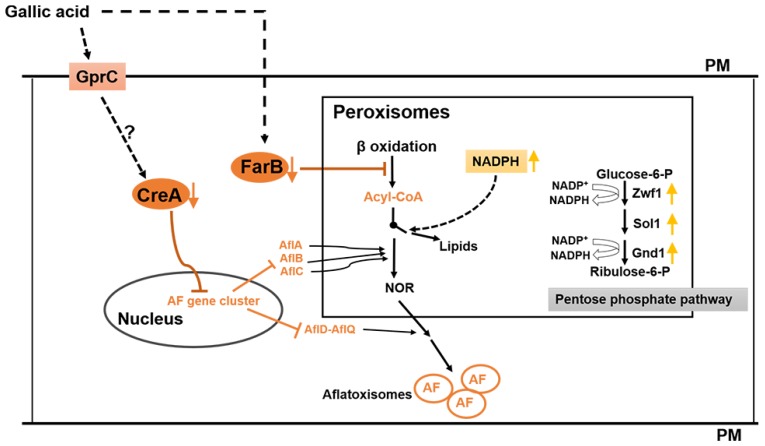
Hypothetical mechanism of gallic acid (GA) inhibition of aflatoxin biosynthesis in *A. flavus*. GA inhibits (i) the expression of *creA*, which leads to the downregulation of the aflatoxin (AF) cluster genes, simultaneously GA (ii) reduces the peroxisomal fatty acids β-oxidation level by diminishing the expression of *farB*, resulting in decreasing the aflatoxin precursor acyl-CoA pools, and (iii) increasing the NADPH level via the pentose phosphate pathway, which favors lipid synthesis over aflatoxin biosynthesis in *A. flavus*. PM: Plasma membrane. The orange downward arrows indicate the expression of the genes that were significantly downregulated by GA treatment, the yellow upward arrows indicate the higher activity of the pentose phosphate pathway, which leads to increasing NADPH pools. Dotted lines indicate possible connecting pathways and solid lines indicate known pathways.

Aflatoxin biosynthesis is also closely associated with carbohydrate catabolism [41]. For example, glucose, sucrose, and fructose induced aflatoxin formation, while peptone and lactose did not support aflatoxin production [28,42]. Abdollahi et al. [28] suggested that the aflatoxin formation induced by these carbon sources was as a feedback regulation of the elevated energy state which was caused by the utilization of a readily-metabolizable carbohydrate. Microorganisms prefer utilizing the most favored carbon source with the mechanism of carbon repression, and the transcription factor CreA is a well-known as carbon repression regulator in *Aspergillus* [31]. In *Aspergillus nidulans*, CreA can regulate carbon utilization genes via directly binding to the consensus sequence 5′-SYGGRG-3′ in the promoter of these genes [32], and is critical for normal growth on carbon, nitrogen, and lipid sources [43]. The CreA consensus binding sites have been found in the promoter regions of 14 assigned aflatoxin genes (Table 3), and the expression of almost all the aflatoxin genes (except the regulator genes *aflR* and *aflS*) and the *creA* gene, AFLA_134680, were significantly suppressed by the 0.8% (*w*/*v*) GA treatment (Figure 4, Table 2). To confirm the hypothesis that CreA was involved in aflatoxin gene regulation, we deleted *creA* in *A. flavus* and the ∆*creA* mutants were unable to produce the mycotoxin. The qRT-PCR result showed that the expression of the aflatoxin genes were significantly down regulated in the ∆*creA* mutants compared to WT, which is consistent with an identical role for this protein in aflatoxin biosynthesis in another strain of *A. flavus*, CA14 [18]. Whether the regulation is direct via CreA binding of individual *afl* genes or indirect is still to be determined. In *A. flavus*, GprC was considered as a carbon sensing receptor [38] which may mediate the expression of carbon utilization genes. As the GprC encoding gene was significantly inhibited by 0.8% (*w*/*v*) GA treatment, we propose that GA may depress *creA* expression through GprC signaling. Taking these results together, we suggest that GA inhibits aflatoxin biosynthesis in *A. flavus* via modulating the expression of the FarB and CreA pathways, a hypothetical mechanism proposed in Figure 7.

## 4. Materials and Methods

### 4.1. Fungal Strain and Culture Conditions

*Aspergillus flavus* NRRL3357 [44], TJES20.1 (∆*ku70*, ∆*argB*::*A. fumigatus pyrG*, *pyrG*-) [45], and TJW149.27 (∆*ku70*::*A. parasiticus pyrG*, *pyrG*-) were used in this research. A total of 10^7^ *A. flavus NRRL3357* spores were inoculated into 30 mL PDB (BD Difco) with different concentrations (0, 0.2%, 0.5%, 0.8%, and 1% *w*/*v*) of GA, cultured at 30 °C, 200 rpm for aflatoxin analysis or mycelia weight measurement. A total of 10^3^ spores were inoculated on PDA (BD Difco) medium with different concentrations (*w*/*v*) of GA and cultured at 30 °C for diameter determination. TJES20.1 was used for deleting *creA* gene in *A. flavus*, and TJW149.27 was used as control for *creA* mutants.

### 4.2. Aflatoxin Analysis and Measuring the A. flavus Colony Diameters and Mycelia Weights

Aflatoxin was extracted from 200 µL culture medium and analyzed by modified thin-layer chromatography (TLC) [46]. A total of 10^3^ *A. flavus* spores were point-inoculated on PDA medium with different concentrations of GA, the plates were cultured at 30 °C incubator, and the diameters were measured on different culture days. To investigate the effect of GA on *A. flavus* mycelia growth, 10^7^ *A. flavus* spores were inoculated into 30 mL PDB medium with or without 1% (*w*/*v*) GA, and cultured at 30 °C, 200 rpm. The mycelia were collected by using vacuum filtration and measured by analytical balance (0.0001 g) after drying in the oven.

### 4.3. RNA Extraction and cDNA Library Preparation for RNA-Seq Analysis

Triplicate cultures of 10^7^ *A. flavus* spores were grown in PDB medium with 0, 0.2%, and 0.8% (*w*/*v*) GA, respectively, at 30 °C, 200 rpm, harvested after 24 h, and ground in a mortar and pestle under liquid nitrogen. TRIzol reagent (Invitrogen, Carlsbad, CA, USA) was used to extract total RNA from ground mycelia, according to the manufacturer’s instructions. Library construction and RNA quality analysis for all nine samples were conducted by Annoroad (Beijing, China). The Agilent 2100 bioanalyzer with Agilent RNA 6000 Nano Kit (Agilent Technologies, Santa Clara, CA, USA) was used to assess the integrity and purity of the RNA samples. All samples had an RNA Integrity Number (RIN) with more than 8.0. The cDNA library construction was conducted as follows: The mRNA was purified by using Oligo (dT)-attached magnetic beads and fragmented into small pieces by Fragmentation Buffer. The fragmented mRNA was copied into first strand cDNA using random hexamer primers, then the dNTPs, RNase H, and DNA polymerase I were added into the reactions to get the second strand cDNA. These cDNA fragments were purified with QIAQuick PCR kit and eluted with elution buffer, then these cDNA fragments were treated through an end repair process, an addition of a single ‘A’ base, and then ligation of the sequencing adapters. Finally, the cDNA library was sequenced on the Illumina HiSeq platform.

### 4.4. Data Analysis and Normalizing the Expression Levels of Genes

After removing the reads adapters, low quality reads, and reads with more than 5% N, the remaining reads were mapped to the *A. flavus* NRRL3357 genome (https://www.ncbi.nlm.nih.gov/nuccore/AAIH00000000.2) by using TopHat v2.0.12 [47] and Bowtie2 [48] software. The Fragments per Kilobase per Million Mapped Fragments (FPKM) [19] was used to normalize the expression levels of the genes in these sequenced samples. The mean value of the count number of each gene from the three sequenced samples were analyzed by DEseq software [20] for genes’ different expression analysis, the genes from each compared sample with |log2Ratio| ≥ 1 and *q* < 0.05 were considered as significantly differentially expressed between these compared samples. GO analysis was done by Blast2GO software. The transcriptomic profile of the detectable genes in this research can be found in Table S3.

### 4.5. creA Mutant Construction and qRT-PCR Analysis

All primers used for *creA* mutant construction are listed in Table S7. The homologous recombination technology was used to create a *creA* mutant [45]. All PCR-generated flanks ranged in size around 1.5 kb, *argB* with its own promotor and terminator from *A. flavus* and wild type genomic DNA was used as the marker gene. The transformation construct for *creA* mutant was made by double joint PCR [49] and transformed into the parental strain TJES20.1 (∆*ku70*, ∆*argB*::*Aspergillus fumigatus pyrG*, *pyrG*-). Transformation of fungal strains was carried out according to the protocol of Szewczyk et al. [50], with some modifications that can be found in Zhao et al. [51]. The *creA* mutants were confirmed by PCR and qRT-PCR (Figure S1A,B), and aflatoxin synthesis of *creA* mutants (Figure S1C) was analyzed by TLC, the primers for qRT-PCR can be found in Table S7. A total of 10^7^ spores of WT, ∆*creA*-1, and ∆*creA*-2 were inoculated into 30 mL PDB medium, and cultured at 200 rpm, 30 °C for 3 days, and the mycelia were collected for RNA extraction. RNA was extracted by Trizol (Invitrogen, Carlsbad, CA, USA) following the manufacturer’s instructions. RNA quality and quantity were measured by NanoVue plus (GE Healthcare, Buckinghamshire, UK). Reverse transcription was carried out by HiScriptIIQ RT SuperMix (Vazyme, Nanjing, China) for qRT-PCR.

### 4.6. Antioxidant Enzymatic Activities and the Lipid Oxidation Level Measurement

In order to observe the impact of GA on the antioxidant system activities in *A. flavus*, superoxide dismutase (SOD), catalase (CAT), and the content of Malondialdehyde (MDA) were performed on 24 h cultures, following the same culture conditions of RNA isolation. 

Around 300 mg mycelium were suspended in 800 µL PBS buffer and homogenized by Tissuelyser^®^ (Jingxin industrial development Co., Shanghai, China). Then samples were centrifuged at 12,000 rpm for 10 min at 4 °C, the supernatants were used for measuring the enzymes activities, MDA content, and protein concentrations. The catalase assay kit (S0051) and total superoxide dismutase assay kit with WST-8 (S0101) were used to measure the enzymes activities, the MDA content was determined using lipid peroxidation MDA assay kit (S0131), and protein concentrations were measured using an enhanced BCA protein assay kit (P0010). All these measurements were conducted according to manufacturer’s instructions, and these kits were bought from Beyotime Biotechnology (Beijing, China). The enzymatic activities and MDA content were normalized according to their protein concentrations. 

### 4.7. Statistical Analysis

All the wet-lab experiments were conducted in triplicate. The GraphPad Prism software (La Jolla, CA, USA) was used for statistical analysis. Statistically significant differences were determined by an unpaired Student’s *t*-test with a two-tailed distribution and *p* < 0.05. The error bars in all figures indicate the standard error of the mean.

## Figures and Tables

**Table 1:** Summary of the significantly differentially expressed genes among the three samples.

Sample Name	P02_P	P08_P
Up	271	764
Down	264	1276
Total	535	2040

P02_P: 0.2% (*w*/*v*) GA treated samples (P02) compared with untreated samples (P), P08_P: 0.8% (*w*/*v*) GA treated samples (P08) compared with untreated samples (P). Up/Down: Genes’ expression was significantly upregulated/downregulated in the samples before the dash, Total: All the significantly differentially expressed genes between the compared samples.

**Table 2:** Effect of gallic acid (GA) treatment on the expression of the transcription factor genes and primary metabolites related genes in *Aspergillus flavus.*

	P02-P ^a^				P08-P ^b^			
Gene Name	Log2 Ratio ^c^	*p* Value	Up/Down	Significant ^e^	Log2 Ratio ^d^	*p* Value	Up/Down	Significant ^e^
*veA* (AFLA_066460)	−0.4470	0.6546	down	no	−0.3915	2.64 × 10^−^^1^	down	no
*laeA* (AFLA_033290)	−0.2987	0.8134	down	no	−0.6969	1.71 × 10^−^^2^	down	no
*velB* (AFLA_081490)	0.9331	0.1439	up	no	0.0635	9.35 × 10^−^^1^	up	no
*brlA* (AFLA_082850)	−2.5580	0.0010	down	yes	−2.3343	9.88 × 10^−^^6^	down	yes
*NsdC* (AFLA_131330)	1.6888	0.0091	up	yes	1.1464	3.45 × 10^−^^5^	up	yes
*creA* (AFLA_134680)	−0.3367	0.8277	down	no	−1.1486	9.15 × 10^−^^8^	down	yes
*farB* (AFLA_012010)	−0.7799	0.4354	down	no	−1.5190	4.81 × 10^−^^5^	down	yes
*foxA* (AFLA_041590)	−0.5139	0.6420	down	no	−1.1589	1.71 × 10^−^^5^	down	yes
*AcuJ* (AFLA_135240)	−0.8148	0.2928	down	no	−1.2998	1.85 × 10^−^^5^	down	yes
*PexK* (AFLA_036410)	−0.7519	0.3792	down	no	−0.8524	2.72 × 10^−^^4^	down	no
*zwf1* (AFLA_086620)	0.6412	0.4931	up	no	0.7590	6.7 × 10^−^^3^	up	no
*sol3* (AFLA_080390)	0.2110	0.8837	up	no	1.0991	1.0 × 10^−^^4^	up	yes
*gnd1* (AFLA_036840)	0.3718	0.7936	up	no	0.9072	1.3 × 10^−^^3^	up	no

^a^: 0.2% (*w*/*v*) GA-treated samples (P02) compared with the untreated samples (P). ^b^: 0.8% (*w*/*v*) GA-treated samples (P08) compared with the untreated samples (P). ^c^: The mean value of the count number of each gene in P02/in P. ^d^: The mean value of the count number of each gene in P08/in P. ^e^: The genes with |Log2 ratio| ≥ 1 and *p* value < 0.05 were considered as significantly differentially expressed, described as ‘yes’, otherwise described as ‘no’.

**Table 3:** Presence of the CreA consensus binding motif (5′-SYGGRG-3′) in the aflatoxin biosynthesis genes promotor region.

Gene Name	CreA Binding Motif (5′-SYGGRG-3′) ^a^	Position Relative to Start Codon	Description
AFLA_139190	GTGGAG	−361	*aflK*/*vbs*/VERB synthase
AFLA_139200	CTGGAG	−795	*aflQ*/*ordA*/*ord*-1/oxidoreductase/cytochrome P450 monooxigenase
AFLA_139210	No motif found		*aflP*/*omtA*/*omt*-1/*O*-methyltransferase A
AFLA_139220	GTGGGG	−1201	*aflO*/*omtB*/*dmtA*/*O*-methyltransferase B
AFLA_139230	CTGGGG/CCGGAG/GCGGAG	−355/−398/−301	*aflI*/*avfA*/cytochrome P450 monooxygenase
AFLA_139250	CTGGGG	−238	*aflL*/*verB*/desaturase/P450 monooxygenase
AFLA_139260	CTGGGG/GCGGAG	−365/−810	*aflG*/*avnA*/*ord*-1/cytochrome P450 monooxygenase
AFLA_139280	No motif found		*aflN*/*verA*/monooxygenase
AFLA_139300	No motif found		*aflM*/*ver*-1/dehydrogenase/ketoreductase
AFLA_139310	CTGGGG/GCGGAG	−482/−617	*aflE*/*norA*/*aad*/*adh*-2/NOR reductase/dehydrogenase
AFLA_139320	CCGGGG/CTGGGG	−387/−299	*aflJ*/*estA*/esterase
AFLA_139330	GTGGGG/GCGGAG	−488/−58	*aflH*/*adhA*/short chain alcohol dehydrogenase
AFLA_139340	GTGGAG/CCGGGG	−737/−762	*aflS*/pathway regulator
AFLA_139360	GTGGGG/CCGGAG/CTGGAG	−369/−895/−276	*aflR*/*apa*-2/*afl*-2/transcription activator
AFLA_139370	CTGGAG/CTGGGG	−450/−819	*aflB*/fas-1/fatty acid synthase beta subunit
AFLA_139380	GTGGAG	−116	*aflA*/*fas*-2/*hexA*/fatty acid synthase alpha subunit
AFLA_139390	No motif found		*aflD*/*nor*-1/reductase
AFLA_139410	GTGGCG	−763	*aflC*/*pksA*/*pksL1*/polyketide synthase
AFLA_139440	No motif found		*aflF*/*norB*/dehydrogenase

^a^: 5′-SYGGRG-3′, S: C + G, Y: C + T, R: A + G.

**Table 4:** Effect of GA on the expression of the antioxidant-related genes in *A. flavus*.

	P02-P ^a^				P08-P ^b^			
Gene Name	Log2 Ratio ^c^	*p* Value	Up/Down	Significant ^e^	Log2 Ratio ^d^	*p* Value	Up/Down	Significant ^e^
*ppoC* (AFLA_030430)	−2.0268	0.0003	down	yes	−1.4056	7.25 × 10^−2^	down	no
*gprC* (AFLA_074150)	−1.4207	0.0703	down	no	−1.5934	4.14 × 10^−6^	down	yes
*gprK* (AFLA_009790)	−1.2764	0.3936	down	no	−2.1266	9.73 × 10^−4^	down	yes
*gprM* (AFLA_075000)	−2.0079	0.0069	down	yes	−1.4338	3.74 × 10^−2^	down	yes
glutathione S-transferase (AFLA_016400)	0.1570	0.9701	up	no	1.1480	1.20 × 10^−4^	up	yes
glutathione S-transferase (AFLA_023740)	0.4667	0.6766	up	no	1.2355	2.13 × 10^−3^	up	yes
glutathione S-transferase (AFLA_087240)	0.8037	0.3272	up	no	2.0188	1.07 × 10^−12^	up	yes
AhpC/TSA family thioredoxin peroxidase (AFLA_097940)	1.1384	0.0655	up	no	1.2150	4.41 × 10^−5^	up	yes
thioredoxin reductase Trr1/Trr2 (AFLA_051770)	0.8950	0.2535	up	no	1.0196	3.79 × 10^−2^	up	yes

^a^: 0.2% (*w*/*v*) GA-treated samples (P02) compared with the untreated samples (P). ^b^: 0.8% (*w*/*v*) GA-treated samples (P08) compared with the untreated samples (P). ^c^: The mean value of the count number of each gene in P02/in P. ^d^: The mean value of the count number of each gene in P08/in P. ^e^: The genes with |Log2 ratio| ≥ 1 and *p* value < 0.05 were considered as significantly differentially expressed, described as ‘yes’, otherwise described as ‘no’.

## Supplementary Materials

The following are available online at http://www.mdpi.com/2072-6651/10/7/270/s1, Table S1: The inhibition rate and significance analysis of gallic acid on *A. flavus* growth. Table S2: Summary of the RNA-Seq data. Table S3: The transcriptomic profile of the detectable genes and the significantly differentially expressed genes. Table S4: The backbone genes of the 56 secondary metabolism clusters. Table S5: The genes enriched in peroxisome and fatty acid β-oxidation based on the GO analysis. Table S6: The GO data in this research. Table S7: Primers used to create *creA* mutants and for qRT-PCR analysis. Figure S1: Confirmation of the *creA* mutants and the aflatoxin formation in *creA* mutants.
