# Supplementary material for: The Antioxidant Gallic Acid Inhibits Aflatoxin Formation in Aspergillus flavus by Modulating Transcription Factors FarB and CreA
Source: Toxins (Basel). 2018 Jul 3;10(7):270. doi: 10.3390/toxins10070270 (PMC6071284; doi:10.3390/toxins10070270)
Supplement: Supplementary file 1 [file toxins-10-00270-s001.zip › toxins-321880 SPM for proofreading-XZ.docx]

Supplementary Materials: The Antioxidant Gallic Acid Inhibits Aflatoxin Formation in *Aspergillus Flavus* by Modulating Transcription Factors FarB and CreA

Xixi Zhao, Qing-Qing Zhi, Jie-Ying Li, Nancy P. Keller and Zhu-Mei He


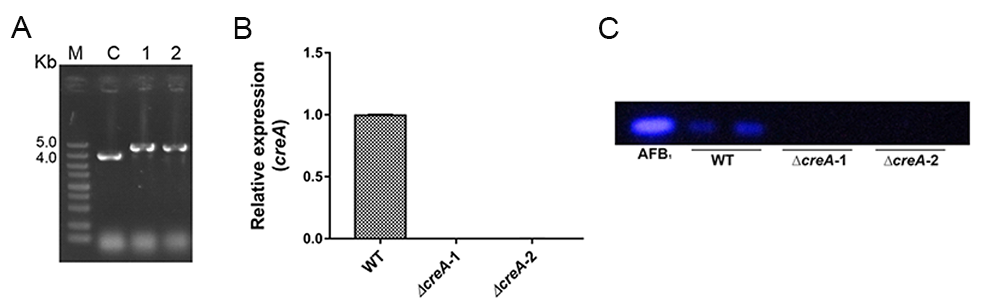


**Figure S1.** Confirmation of *creA* mutants (**A** and **B**) and the aflatoxin formation in *creA* mutants (**C**). (**A**)**.** The primers *creA*-NF and *creA*-NR was used to confirm the *creA* mutants, the size of *creA*-NF and *creA*-NR from WT is around 3.8 kb, from *creA* deletion mutants is around 4.5 kb. (**B).** Primers *creA*-F and *creA*-N was used for qRT-PCR to confirm the *creA* deletion mutants ∆*creA-1* and ∆*creA-2*. (**C).** 10^7^ spores of WT, ∆*creA-1* and ∆*creA-2* strains were inoculated into 30 mL PDB, and cultured at 200 rpm, 30 °C for 3 days, 200 µL culture medium was used for aflatoxin TLC analysis.

**Table S1.** The inhibition rate and significance analysis of gallic acid on *A. flavus* growth

|  | **PDA** | **PDA+0.2% GA** | | **PDA+0.5% GA** | | **PDA+0.8% GA** | | **PDA+1% GA** | |
| --- | --- | --- | --- | --- | --- | --- | --- | --- | --- |
|  | Dc  (cm) | inhibition rate^#^ | Log2  (Ds/Dc*） | inhibition rate | Log2  (Ds/Dc） | inhibition rate | Log2  (Ds/Dc） | inhibition rate | Log2  (Ds/Dc） |
| 3 d^a^ | 2.75 | 0.09 | -0.14 | 0.15 | -0.24 | 0.21 | -0.33 | 0.24 | -0.40 |
| 4 d | 3.82 | 0.08 | -0.12 | 0.15 | -0.23 | 0.19 | -0.30 | 0.21 | -0.34 |
| 5 d | 4.62 | 0.06 | -0.10 | 0.14 | -0.22 | 0.18 | -0.29 | 0.22 | -0.36 |
| 6 d | 5.47 | 0.07 | -0.10 | 0.15 | -0.23 | 0.19 | -0.31 | 0.21 | -0.34 |
| 7 d | 6.23 | 0.07 | -0.11 | 0.16 | -0.25 | 0.19 | -0.30 | 0.21 | -0.35 |
| 10 d | 8.00 | 0.04 | -0.06 | 0.10 | -0.15 | 0.14 | -0.21 | 0.16 | -0.26 |

^#^: inhibition rate=1-Ds/Dc. *: Ds: the mean value of gallic acid treated *A. flavus* colonies diameter. Dc: the mean value of the untreated *A. flavus* colonies diameter with the same culture days. ^a^: days.

**Table S2.** Summary of the RNA-Seq data

| **Sample** | **P1** | **P2** | **P3** | **P02-1** | **P02-2** | **P02-3** | **P08-1** | **P08-2** | **P08-3** |
| --- | --- | --- | --- | --- | --- | --- | --- | --- | --- |
| Raw Reads Number | 23,898,280 | 22,778,644 | 23,273,248 | 23,292,774 | 23,976,760 | 22,857,152 | 20,548,080 | 22,896,190 | 24,414,006 |
| Clean Reads Number | 23,118,022 | 22,133,478 | 22,624,608 | 22,585,384 | 23,340,760 | 22,191,008 | 19,892,462 | 22,142,460 | 23,708,028 |
| Clean Reads Rate (%) | 96.73 | 97.17 | 97.21 | 96.96 | 97.35 | 97.09 | 96.81 | 96.71 | 97.11 |
| Mapped Reads | 20,770,075 | 19,955,585 | 20,593,157 | 20,194,237 | 20,894,924 | 17,143,917 | 18,172,737 | 19,738,124 | 21,416,603 |
| Mapping Rate (%) | 0.8984 | 0.9016 | 0.9102 | 0.8941 | 0.8952 | 0.7726 | 0.9135 | 0.8914 | 0.9033 |
| UnMapped Reads | 2,347,947 | 2,177,893 | 2,031,451 | 2,391,147 | 2,445,836 | 5,047,091 | 1,719,725 | 2,404,336 | 2,291,425 |
| MultiMap Reads | 72,026 | 78,501 | 55,283 | 80,440 | 74,028 | 270,335 | 42,123 | 94,076 | 52,264 |
| MultiMap Rate (%) | 0.0031 | 0.0035 | 0.0024 | 0.0036 | 0.0032 | 0.0122 | 0.0021 | 0.0042 | 0.0022 |
| UniqueMap Reads | 18,350,102 | 17,699,191 | 18,506,423 | 17,722,650 | 18,375,060 | 11,826,491 | 16,410,889 | 17,239,712 | 19,072,914 |
| UniqueMap Rate (%) | 0.8834875 | 0.8869292 | 0.8986686 | 0.8776093 | 0.8794031 | 0.689836 | 0.9030499 | 0.873422 | 0.890567 |

P1, P2, P3: triplicates of the untreated *A. flavus* samples. P02-1, P02-2, P02-3: triplicates of the 0.2% (w/v) gallic acid treated *A. flavus* samples. P08-1, P08-2, P08-3: triplicates of the 0.8% (w/v) gallic acid treated *A. flavus* samples.
